# Hydrogen Sulfide Mediates K^+^ and Na^+^ Homeostasis in the Roots of Salt-Resistant and Salt-Sensitive Poplar Species Subjected to NaCl Stress

**DOI:** 10.3389/fpls.2018.01366

**Published:** 2018-09-19

**Authors:** Nan Zhao, Huipeng Zhu, Huilong Zhang, Jian Sun, Jinchi Zhou, Chen Deng, Yuhong Zhang, Rui Zhao, Xiaoyang Zhou, Cunfu Lu, Shanzhi Lin, Shaoliang Chen

**Affiliations:** ^1^Beijing Advanced Innovation Center for Tree Breeding by Molecular Design, College of Biological Sciences and Technology, Beijing Forestry University, Beijing, China; ^2^Public Analysis and Testing Center, Beijing Forestry University, Beijing, China; ^3^Beijing Dabeinong Technology Group Co., Ltd., Beijing, China; ^4^College of Life Science, Jiangsu Normal University, Xuzhou, China

**Keywords:** NaHS, NaCl, root, ion flux, *Populus euphratica*, *Populus popularis*, NMT

## Abstract

Non-invasive micro-test techniques (NMT) were used to analyze NaCl-altered flux profiles of K^+^, Na^+^, and H^+^ in roots and effects of NaHS (a H_2_S donor) on root ion fluxes in two contrasting poplar species, *Populus euphratica* (salt-resistant) and *Populus popularis* (salt-sensitive). Both poplar species displayed a net K^+^ efflux after exposure to salt shock (100 mM NaCl), as well as after short-term (24 h), and long-term (LT) (5 days) saline treatment (50 mM NaCl, referred to as salt stress). NaHS (50 μM) restricted NaCl-induced K^+^ efflux in roots irrespective of the duration of salt exposure, but K^+^ efflux was not pronounced in data collected from the LT salt stress treatment of *P. euphratica*. The NaCl-induced K^+^ efflux was inhibited by a K^+^ channel blocker, tetraethylammonium chloride (TEA) in *P. popularis* root samples, but K^+^ loss increased with a specific inhibitor of plasma membrane (PM) H^+^-ATPase, sodium orthovanadate, in both poplar species under LT salt stress and NaHS treatment. This indicates that NaCl-induced K^+^ loss was through depolarization-activated K^+^ channels. NaHS caused increased Na^+^ efflux and a corresponding increase in H^+^ influx for poplar roots subjected to both the short- and LT salt stress. The NaHS-enhanced H^+^ influx was not significant in *P. euphratica* samples subjected to short term salt stress. Both sodium orthovanadate and amiloride (a Na^+^/H^+^ antiporter inhibitor) effectively inhibited the NaHS-augmented Na^+^ efflux, indicating that the H_2_S-enhanced Na^+^ efflux was due to active Na^+^ exclusion across the PM. We therefore conclude that the beneficial effects of H_2_S probably arise from upward regulation of the Na^+^/H^+^ antiport system (H^+^ pumps and Na^+^/H^+^ antiporters), which promote exchange of Na^+^ with H^+^ across the PM and simultaneously restricted the channel-mediated K^+^ loss that activated by membrane depolarization.

## Introduction

High salt content in soil leads to plant growth inhibition due to ion toxicity, water shortage, and nutrient imbalances (Zhu, 2001). The maintenance of K^+^ and Na^+^ homeostasis is essential for herbaceous and woody species growing in or adapting to saline environments (Chen et al., 2001, 2002, 2003; Cuin et al., 2003; Ottow et al., 2005; Wang et al., 2006, 2007, 2008; Shabala and Cuin, 2008; Sun et al., 2009a). NaCl-induced K^+^ loss observed in higher plants is mediated by depolarization-activated KORCs (outward rectifying K^+^ channels) and NSCCs (non-selective cation channels; Chen et al., 2007; Shabala and Cuin, 2008; Sun et al., 2009b). The Na^+^/H^+^ antiport system contributes to Na^+^ extrusion and vacuolar compartmentation (Blumwald et al., 2000; Shi et al., 2000; Apse et al., 2003; He et al., 2005). Na^+^ movement is highly dependent on H^+^-ATPase since the H^+^ pumps provide a proton gradient to drive the Na^+^/H^+^ exchange across the plasma and vacuolar membranes (Blumwald et al., 2000; Zhu, 2003; Sun et al., 2009a,b; Ma et al., 2010). Moreover, H^+^-ATPase also inhibits the entry of Na^+^ through NSCCs (Maathuis and Sanders, 2001; Maathuis, 2006).

A variety of stress signals, extracellular ATP, H_2_O_2_, cytosolic [Ca^2+^], and NO, mediate ionic homeostasis under NaCl stress through regulations of the Na^+^/H^+^ antiport system (Zhang et al., 2006; Shi et al., 2007; Sun et al., 2010a,b, 2012; Lu et al., 2013). Evidence increasingly shows that hydrogen sulfide (H_2_S) functions as a molecular signal that also mediates numerous physiological processes. H_2_S has been shown to regulate photosynthesis (Chen et al., 2011), stomatal movement (García-Mata and Lamattina, 2010; Lisjak et al., 2010; Jin and Pei, 2016; Jin et al., 2017), adventitious rooting (Zhang et al., 2009a), and flower senescence (Zhang et al., 2011). The role of H_2_S in stress physiology has thus received growing attention among researchers. Researchers have shown that H_2_S mediates plant adaptive response to boron (Wang et al., 2010), aluminum (Zhang et al., 2010a), copper (Zhang et al., 2008), cadmium (Li et al., 2012; Sun et al., 2013; Qiao et al., 2016), chromium stress (Fang et al., 2016), and general osmotic and drought stresses (Zhang et al., 2009b, 2010b). Interestingly, H_2_S enhanced alfalfa’s ability to tolerate NaCl during seed germination through a nitric oxide pathway (Wang et al., 2012). Under NaCl stress, Wang et al. (2012) showed that NaHS (a H_2_S donor) increased the ratio of K^+^/Na^+^ in root structures of germinated alfalfa seeds. Endogenous H_2_S increased salt tolerance by preventing K^+^ loss and reestablishing redox homeostasis in *Medicago sativa* seedlings (Lai et al., 2014). Janicka et al. (2017) confirmed the involvement of H_2_S as a signaling molecule in the modification of plasma membrane (PM) H^+^-ATPase in salt-exposed cucumber roots. H_2_S likely interacts with NO in to modify antioxidant and redox defense systems in tomato and tobacco plants (da Silva et al., 2017; da-Silva et al., 2018; da-Silva and Modolo, 2017). Furthermore, exogenous H_2_S alleviates salt stress by reducing Na^+^ content in wheat seedlings (Deng et al., 2016). The physiological mechanism by which H_2_S influences K^+^/Na^+^ homeostasis in woody plants, however, remains unclear.

The objectives of present study were to evaluate the roles of H_2_S to K^+^/Na^+^ homeostasis in two poplar species with differing salt tolerances: the salt-resistant *Populus euphratica* and the salt-sensitive *Populus popularis*. We used non-invasive micro-test techniques (NMT) to measure the NaCl-induced flux profiles of K^+^, Na^+^, and H^+^ in the presence and absence of H_2_S. This study also applied both short- and long-term (LT) salt treatments to subjects in order to clarify the effects of H_2_S on salt tolerance. The effects of PM transport inhibitors on ion flux were characterized in order to test specific physiological mechanisms linking NaHS to K^+^/Na^+^ homeostasis in tree plants.

## Materials and Methods

### Plant Materials

In April, one-year-old seedlings of *P. euphratica* grown in the Xinjiang Uygur Autonomous Region of China and one-year-old cuttings of *P. popularis* 35–44 (*P. popularis*) grown in the nursery of Beijing Forestry University were planted in individual pots (about 10 L) containing loam soil. These were then raised in a greenhouse. Plant subjects were kept well irrigated and supplemented with full-strength Hoagland’s nutrient solution every 2 weeks. Greenhouse air temperature ranged from 25 to 30°C while relative humidity ranged from 60 to 70%. Plants were subjected to a 12-h photoperiod (7:00–19:00) with photosynthetically active radiation varying from 400 to 800 μmol m^-2^ s^-1^. Plants grew for one month prior to hydroponic culture. Rooted seedlings and cuttings were then collected, washed free of soil, and transferred to 2 L individual porcelain pots containing diluted (25% strength) Hoagland’s nutrient solution. The plants were continuously aerated during the period of hydroponic acclimation. Nutrient solution was renewed every 2 days.

### Treatments Designed for Flux Recordings

#### Series 1

Root samples of *P. euphratica* and *P. popularis* controls were excised, immediately rinsed with re-distilled water, followed by 30 min equilibration in a basic solution [NaCl (0.1 mM), MgCl_2_ (0.1 mM), CaCl_2_ (0.1 mM), and KCl (0.5 mM)] supplemented with or without 25, 50, and 200 μM NaHS (Sigma, St. Louis, MO, United States). The pH of the solution was adjusted to 5.7 with NaOH and HCl. Thereafter, steady K^+^ flux was recorded in the apical region (ca. 300 μm from the root tip) for 10 min before the addition of salt. A salt shock (100 mM NaCl) was applied by adding NaCl stock (0.2 M, pH 6.0 adjusted with NaOH and HCl). Transient ion fluxes were monitored for an additional 40 min. The flux data collected during the first 2–3 min were not included in analysis due to diffusion effects from stock addition (Shabala, 2000). For each dose treatment, NaCl-altered K^+^ kinetics were recorded from at least five individual plants.

#### Series 2

*Populus euphratica* and *P. popularis* plants were subjected to one of the four treatments: control, control plus NaHS (50 μM; Sigma, St. Louis, MO, United States), NaCl (50 mM), and NaCl (50 mM) plus NaHS (50 μM). NaHS acts as a H_2_S donor (Zhao et al., 2001; Wang et al., 2010). The NaCl and NaHS salts were added to the full-strength Hoagland’s nutrient solution. A short-term (ST) saline treatment exposed plants to 50 mM NaCl for 24 h while the LT saline treatment exposed plants to 50 mM NaCl for 5 days. Controls were fertilized but not treated with NaCl or NaHS. In the long term treatment of 5 days, the solutions containing NaHS were renewed daily to ensure its concentration. Ion flux measurements were performed on 2–3 cm of apical root segments. The K^+^, Na^+^, H^+^ fluxes were recorded individually with NMT selective electrodes. In our study, fluxes of K^+^, Na^+^, and H^+^ were measured using roots sampled from the same plants. For each treatment (control, control+NaHS, NaCl, NaCl+NaHS), flux recordings of each target ion (K^+^, Na^+^, and H^+^) were recorded from at least five individual plants after ST and LT salt stress treatment.

#### Series 3

The same three pharmacological agents were applied to LT- and NaHS-treated roots for 30 min (Sun et al., 2009a). These include:

(1) Twenty millimolars TEA (tetraethylammonium chloride, a K^+^ channel blocker);
(2) Five hundred micromolars sodium orthovanadate (a specific inhibitor of PM H^+^-ATPase);
(3) Fifty micromolars amiloride (a Na^+^/H^+^ antiporter inhibitor).

After inhibitor treatment, roots were immersed in the measuring solution for 30 min equilibration prior to flux recordings (Sun et al., 2009a,b, 2010a; Lang et al., 2014). Steady-state flux of K^+^ and Na^+^ were then recorded for 30 min in *P. euphratica* and *P. popularis* roots pretreated with or without inhibitors. Fluxes of K^+^ and Na^+^ were recorded individually using roots sampled from the same plants. For NaCl, NaHS, and pharmacological treatments, flux recordings of each target ion (K^+^ and Na^+^) were recorded from at least five individual plants.

### Flux Recording of K^+^, Na^+^, and H^+^

#### Preparation and Calculation of Microelectrodes

A NMT system (BIO-001A, Younger USA Sci. & Tech. Corp., Amherst, MA, United States) was used to non-invasively measure net K^+^, Na^+^, and H^+^ fluxes in the *P. euphratica* and *P. popularis* root samples. The ion-selective microelectrodes were prepared according to Sun et al. (2009a,b). Prior to flux measurements, K^+^, Na^+^, and H^+^ microelectrodes were, respectively, calibrated in following solutions:

(1) K^+^ calculation series (mM): 0.1, 0.5, and 1.0;
(2) Na^+^ calculation series (mM): 0.1, 0.5, and 1.0;
(3) H^+^ calculation series: pH 5.0, 6.0, and 7.0.

Our experiments used only electrodes with Nerstain slopes >50 mV/decade.

#### Steady-State NMT Measurements

Young roots (3.0 cm) were sampled from control, NaHS, ST, and LT salt stressed poplars (Series 2). The root tips were rinsed with re-distilled water, and then immediately equilibrated for 30 min in the following solutions (Sun et al., 2009a):

(1) K^+^ and H^+^ measuring solutions (mM): NaCl (0.1), MgCl_2_ (0.1)_,_ CaCl_2_ (0.1), and KCl (0.5), pH 5.7 (adjusted with NaOH and HCl);
(2) Na^+^ measuring solutions (mM): NaCl (0.1), MgCl_2_ (0.1), CaCl_2_ (0.1), and KCl (0.1), pH 5.7 (adjusted with NaOH and HCl).

After equilibration, roots samples were transferred to a NMT measuring chamber containing fresh measuring solution (10 mL) and then immobilized at their bases. Ion fluxes were measured at the root meristematic region (300 μm from the tip). Target ion gradients were measured by moving the electrode between two positions close to the root surface (electrode pre-set excursion 30 μM for roots) at a programmed frequency of 0.3–0.5 Hz. DRIREF-2 (World Precision Instruments) was used as the reference electrode. Steady-state fluxes of K^+^, Na^+^, and H^+^ in *P. euphratica* and *P. popularis* root samples were continuously recorded for 30 min. For control, ST, and LT treatment, flux recordings of each target ion were recorded from at least five individual plants.

Long-term and inhibitor-treated roots (Series 3) were also used for steady-state flux recordings as described above. The inhibitors were not included in the refreshed measuring solutions. This was to avoid the potential interference of these pharmacological agents on flux recordings. We have noticed that sodium orthovanadate had clear effect on the Nernstian slopes of the microelectrodes (e.g., Na^+^), although the effects of amiloride and TEA on Na^+^ and K^+^ microelectrodes were much less (Sun et al., 2009a,b).

### Data Analysis

JCal V3.3 developed by Yue Xu^1^^,^^2^ was used to calculate three-dimensional ionic flux rates. All mean data were subjected to standard analysis of variance (ANOVA) methods. Significant differences between means were determined using Duncan’s multiple range test. Unless otherwise stated, differences are considered statistically significant when *P* < 0.05.

## Results

### Effects of NaHS on Transient K^+^ Kinetics With NaCl Shock

**FIGURE 1:**
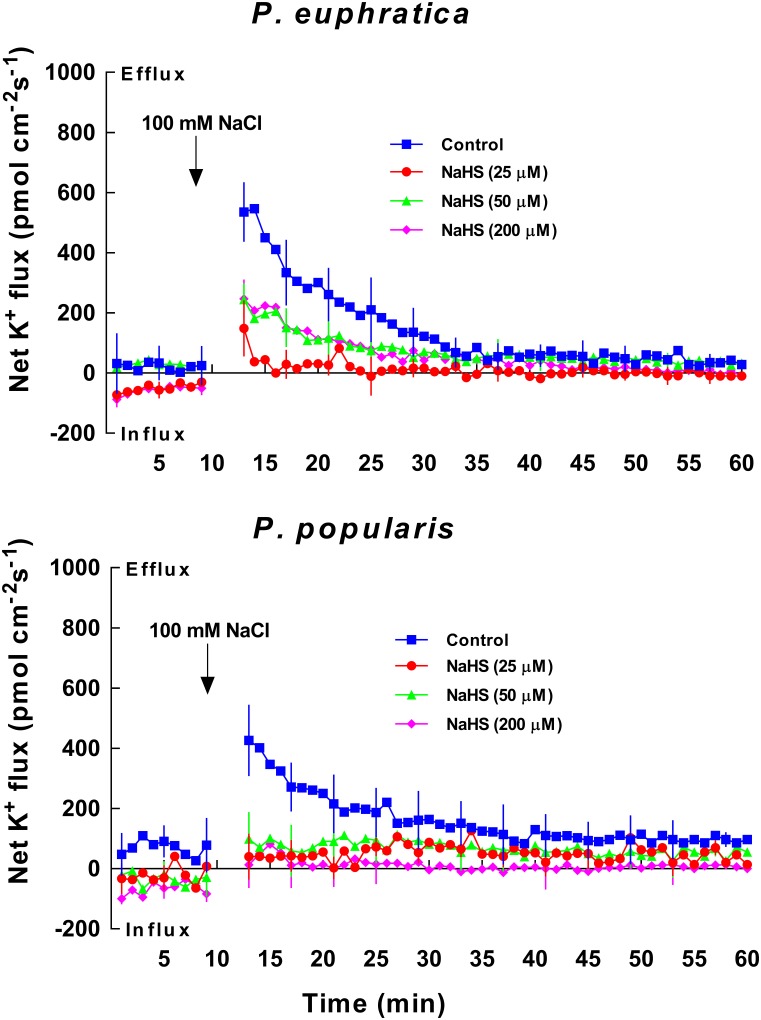
Effects of NaHS on NaCl shock-altered transient K^+^ kinetics within *P. euphratica* and *P. popularis* root samples. Young root samples of *P. euphratica* and *P. popularis* were equilibrated for 30 min in a basic solution [NaCl (0.1 mM), MgCl_2_ (0.1 mM)_,_ CaCl_2_ (0.1 mM), and KCl (0.5 mM)] supplemented with or without 25, 50, and 200 μM NaHS. Thereafter, K^+^ kinetics were recorded at meristems (300 μm from the root tip) for ca. 40 min after NaCl shock. The salt shock (100 mM NaCl) was given by adding acquired amount of NaCl stock (0.2 M, pH 6.0 adjusted with NaOH and HCl) to the measuring solution. Before the NaCl shock, steady-state K^+^ fluxes were recorded for 10 min. Each point is the mean of five individual plants and bars represent the standard error of the mean.

In the two poplar species analyzed, salt-shock induced K^+^ kinetics were recorded in root meristematic zones (ca. 300 μm from the root tip). These typically exhibited a higher K^+^ flux compared to the mature zone and apex (Sun et al., 2009a). Na^+^ kinetics were not examined because of diminished signal/noise ratio from Na^+^ electrodes in a measuring medium with higher Na^+^ (Sun et al., 2009a). After exposure to the 100 mM NaCl solution, *P. euphratica* roots exhibited an instantaneous increase of K^+^ efflux, reaching maximum values of 500 to 600 pmol cm^-2^ s^-1^ (**Figure 1**). Salt-induced K^+^ efflux then showed a gradual decline reaching base levels after 30 min of salt treatment (**Figure 1**). The salt-shocked *P. popularis* roots showed a trend similar to those of the salt-tolerant poplar species (**Figure 1**). Pretreatment with NaHS significantly decreased transient K^+^ efflux induced by salt shock in samples from the two poplar species (**Figure 1**). The K^+^ loss inhibition by NaHS was more pronounced in the salt-sensitive *P. popularis* samples where NaHS exhibited similar inhibitory effects at all test concentrations, (i.e., 25, 50, 200 μM; **Figure 1**). We adopted a mid-range concentration of 50 μM NaHS for further experiments. This concentration generated clear inhibition of salt-stimulated K^+^ efflux in both species analyzed.

### Effects of NaHS on Steady-State Ion Fluxes Under Short-Term and Long-Term NaCl Stress

Steady-state K^+^, Na^+^, and H^+^ fluxes were examined within root meristems after (ST, 24 h) and (LT, 5 days) exposure to NaCl and NaHS. Samples from different species differed in terms of their ion flux response to agonist and salt treatments (see below).

#### K^+^ Flux

**FIGURE 2:**
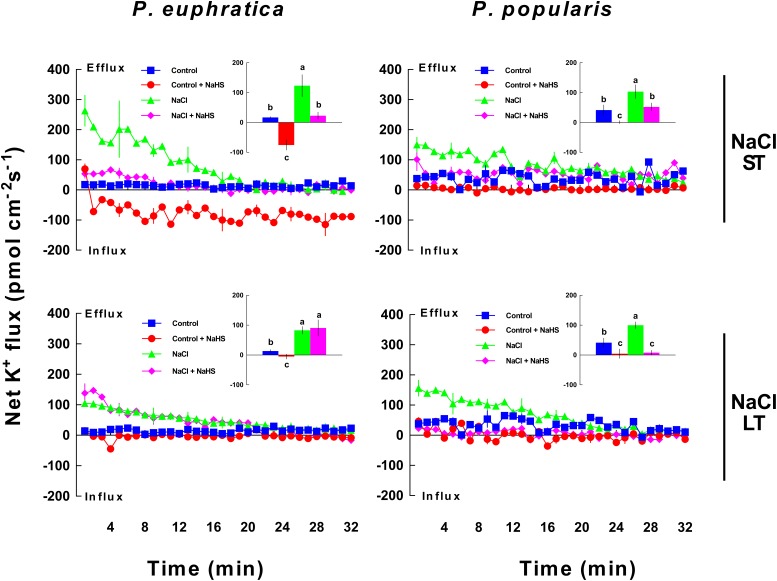
Effect of short-term (ST) and long-term (LT) NaCl treatment on K^+^ fluxes within *P. euphratica* and *P. popularis* roots. Steady-state fluxes were measured at meristems (300 μm from the root tip) for ca. 30 min. Plants were treated with short-term (24 h) and long-term (5 days) NaCl (50 mM) supplemented with or without NaHS (50 μM). Control plants were well fertilized but not given additional NaCl or NaHS. Inlays show the mean flux rates of K^+^ over the first 10 min. Each column shows mean values measured from five individual plants and bars representing the standard error of the mean. Columns labeled with different letters (a, b, and c) denote significant difference at *P* < 0.05.

Short-term salt stress caused a significant net K^+^ efflux in roots of *P. euphratica* and *P. popularis*, although K^+^ flux rates varied between the two poplar species (**Figure 2**). The NaHS (50 μM) markedly reduced salt-induced K^+^ efflux by 49.4–81.6% in ST salt stressed plants of both species (**Figure 2**). Under LT salt stress, the NaHS inhibition of K^+^ loss was evident in NaCl-treated *P. popularis* samples but not detected in the salt-resistant *P. euphratica* samples (**Figure 2**). For *P. popularis*, NaHS caused a greater reduction in K^+^ loss in LT-stressed roots than in ST-stressed roots (**Figure 2**). NaHS also apparently lowered K^+^ efflux in control samples from *P. popularis* or even shifted to a net influx in *P. euphratica* roots (no-salt treatment; **Figure 2**).

#### Na^+^ Flux

**FIGURE 3:**
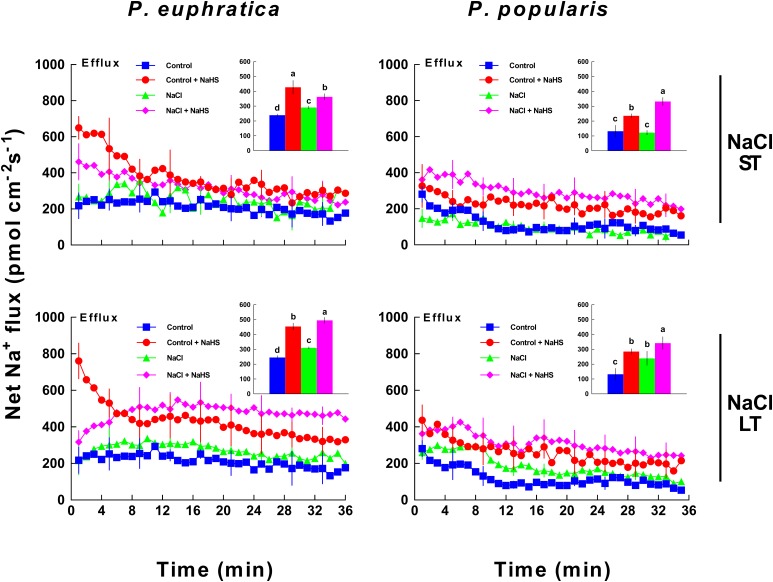
Effect of short-term (ST) and long-term (LT) NaCl treatment on Na^+^ fluxes within *P. euphratica* and *P. popularis* roots. Steady-state fluxes were measured in meristems (300 μm from the root tip) for ca. 30 min. Plants were treated with short-term (24 h) and long-term (5 days) NaCl (50 mM) supplemented with or without NaHS (50 μM). Control plants were well fertilized but not given additional NaCl or NaHS. Inserted sections show the mean flux rates of Na^+^ over the first 10 min. Each column shows mean values measured from five individual plants and bars representing the standard error of the mean. Columns labeled with different letters (a, b, c, and d) denote significant difference at *P* < 0.05.

Steady-state Na^+^ fluxes were measured in roots of the two poplar species following ST (24 h) and LT (5 days) NaCl and NaHS treatments. Relative to control samples, ST salt stress resulted in an increased Na^+^ efflux in *P. euphratica* roots, but not in *P. popularis* roots (**Figure 3**). NaHS (50 μM) applications also markedly enhanced the Na^+^ efflux in *P. euphratica* and *P. popularis* roots regardless of control and salt treatment (**Figure 3**). Na^+^ stimulation by NaHS was more pronounced in samples of the salt-sensitive *P. popularis*, in which Na^+^ efflux increased by 170.1% as compared to the 24.6% increase observed from *P. euphratica* samples (**Figure 3**).

A similar trend was observed under LT salt stress. NaHS stimulated Na^+^ efflux in control and salt stressed root samples of both poplar species, although *P. euphratica* samples typically displayed higher flux rates within the measured apical region (**Figure 3**).

#### H^+^ Flux

**FIGURE 4:**
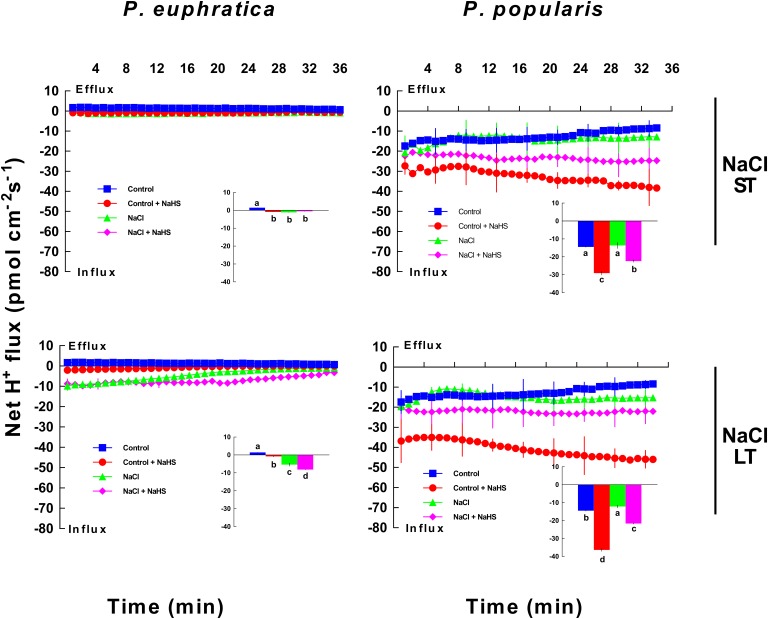
Effect of short-term (ST) and long-term (LT) NaCl treatment on H^+^ fluxes within *P. euphratica* and *P. popularis* roots. Steady-state fluxes were measured within root meristems (300 μm from the root tip) for ca. 30 min. Plants were treated with short-term (24 h) and long-term (5 days) NaCl (50 mM) supplemented with or without NaHS (50 μM). Control plants were well fertilized but not given additional NaCl or NaHS. Inlays show the mean flux rates of H^+^ over the first 10 min. Each column shows mean values measured from five individual plants with bars representing the standard error of the mean. Columns labeled with different letters (a, b, c, and d) denote significant difference at *P* < 0.05.

Root tip control samples of *P. euphratica* exhibited a slight H^+^ efflux that differed from the evident influx observed in *P. popularis* control samples (**Figure 4**). ST salt stress caused an expected shift in H^+^ efflux toward influx in samples from the salt-resistant poplar species (**Figure 4**). However, root tip control samples of *P. popularis* continued to exhibit a net H^+^ influx, which was not significantly altered by ST salt stress treatments (**Figure 4**). This finding is consistent with previous results (Sun et al., 2009a). NaHS (50 μM) significantly increased H^+^ influx in *P. popularis* with a more pronounced effect in controls than in stressed roots (**Figure 4**). However, NaHS enhancement was not significant for ST salt stressed *P. euphratica* samples (**Figure 4**).

Under LT salt stress, NaHS enhanced H^+^ influx in root samples of both species (**Figure 4**). A similar effect was also observed for control samples, where NaHS caused the expected shift in H^+^ efflux to influx in the salt-resistant poplar species, while it enhanced H^+^ influx in the salt-sensitive poplar species (**Figure 4**). For *P. popularis*, the effect of NaHS on H^+^ influx was more pronounced in no-salt roots compared to LT-stressed ones, similar to the findings in the ST treatment (24 h, **Figure 4**).

### Effects of PM Inhibitors on Root K^+^ and Na^+^ Fluxes Under NaHS and LT Salt Stress Treatments

#### K^+^ Flux

**FIGURE 5:**
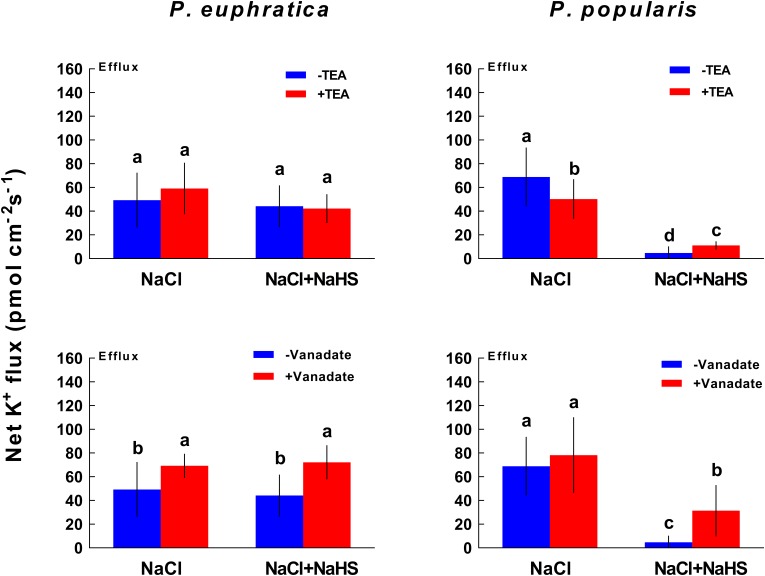
Effect of TEA or sodium orthovanadate on K^+^ fluxes in long-term NaCl-treated roots of *P. euphratica* and *P. popularis*. Prior to flux measurements, salinized roots were subjected to TEA (20 mM) or sodium orthovanadate (500 μM) treatments for 30 min. Steady state K^+^ fluxes were then monitored for another 30 min. Each column shows mean values from five individual plants and bars representing the standard error of the mean. Columns labeled with different letters (a, b, and c) denote significant difference at *P* < 0.05.

In our study, TEA was employed to block the PM K^+^ channels while sodium orthovanadate was used to inhibit H^+^-ATPase in the PM (Sun et al., 2009b; Lu et al., 2013). Inhibitor experiments indicated that the K^+^ channel blocker (TEA) did not significantly inhibit the salt-induced K^+^ efflux in the LT salt stressed *P. euphratica* root samples, regardless of the presence or absence of NaHS (**Figure 5**). In the roots of the salt-sensitive poplar species, TEA reduced K^+^ efflux caused by NaCl (27.0%, **Figure 5**). However, TEA inhibition of K^+^ efflux in NaHS-treated root samples was not as pronounced as that observed in root samples not subjected to NaHS treatment (**Figure 5**). In the former samples, NaHS had markedly reduced K^+^ efflux under salt stress (**Figure 5**).

In contrast to TEA, sodium vanadate accelerated K^+^ efflux in LT salt stressed root samples of *P. euphratica* and *P. popularis*, regardless of the presence or absence of NaHS (**Figure 5**). This indicates that NaHS reduced K^+^ efflux via the activated H^+^-ATPase in the PM, which in turn restricted K^+^ loss through inhibiting depolarization-activated KORCs for both species analyzed (Sun et al., 2009b).

#### Na^+^ Flux

**FIGURE 6:**
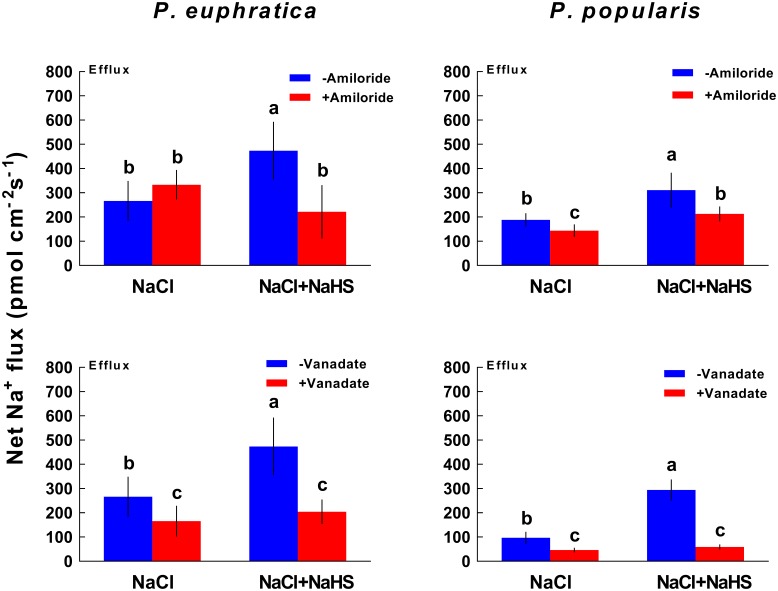
Effect of amiloride or sodium orthovanadate on Na^+^ fluxes in long-term NaCl-treated roots of *P. euphratica* and *P. popularis*. Prior to flux measurements, salinized roots were subjected to amiloride (50 μM) or sodium orthovanadate (500 μM) treatments for 30 min. Steady state Na^+^ fluxes were then monitored for another 30 min. Each column shows mean values from five individual plants and bars representing the standard error of the mean. Columns labeled with different letters (a, b, and c) denote significant difference at *P* < 0.05.

To characterize the effects of PM transport inhibitors on Na^+^ fluxes, we used LT salt-stressed roots of *P. euphratica* and *P. popularis*, which exhibited an evident increase in Na^+^/H^+^ exchange under NaHS treatment (**Figure 3**). **Figure 6** shows NaHS (50 μM) enhanced Na^+^ efflux in LT salt stressed samples. However, amiloride (an inhibitor of Na^+^/H^+^ exchange) or sodium orthovanadate (the inhibitor of PM H^+^-ATPase) significantly inhibited Na^+^ efflux in salinized roots in the presence or absence of NaHS (**Figure 6**). The effects of amiloride were not pronounced in NaCl-treated *P. euphratica* roots (**Figure 6**).

## Discussion

### H_2_S Mediates K^+^/Na^+^ Homeostasis

The capacity to retain K^+^ and Na^+^ homeostasis is critical for poplar species living in saline soils (Chen et al., 2001, 2003). This study showed that H_2_S regulates K^+^/Na^+^ homeostasis in roots of the salt-resistant poplar, *P. euphratica* and those of the salt-sensitive poplar, *P. popularis* (**Figures 1**–**3**). H_2_S treatments similarly increased the ratio of K^+^ to Na^+^ in various herbaceous species, including *M. sativa* (Wang et al., 2012; Lai et al., 2014), *Fragaria* × *ananassa* (Christou et al., 2013), *Hordeum vulgare* (Chen et al., 2015), and *Triticum aestivum* (Deng et al., 2016). Our NMT data revealed that maintenance of root K^+^/Na^+^ homeostasis in NaHS-treated *P. euphratica* and *P. popularis* accounted for greater Na^+^ extrusion and lower K^+^ loss under NaCl stress.

### Na^+^ Homeostasis

NaHS increased net Na^+^ efflux in roots of two poplar species under ST and LT salt stress (**Figure 3**). Moreover, salt stressed *P. euphratica* and *P. popularis* samples exhibited a net influx of H^+^ in NaHS-treated roots (**Figure 4**). In salt-stressed poplars roots, the H_2_S-stimulated H^+^ uptake corresponding to the Na^+^ efflux suggests that Na^+^ extrusion primarily results from active Na^+^/H^+^ antiport across the PM. This finding resembles those reported by our previous studies of salinized roots belonging to *P. euphratica* (Sun et al., 2009a,b; Zhao et al., 2016) and mangrove species such as *Bruguiera gymnorhiza* (Lu et al., 2013), *Kandelia obovata* and *Aegiceras corniculatum* (Lang et al., 2014). Pharmacological evidence showed that inhibitors of PM H^+^-ATPase (sodium orthovanadate) and Na^+^/H^+^ antiporter (amiloride) decreased the H_2_S-stimulated Na^+^ efflux in LT salt stressed plants (**Figure 6**). Collectively, the agonist and antagonist data revealed that the H_2_S enhancement of Na^+^ efflux results from increased activity of Na^+^/H^+^ antiport in NaCl-treated roots of *P. euphratica* and *P. popularis*. The Na^+^/H^+^ antiport system in the PM (Na^+^/H^+^ antiporter and H^+^-ATPase) contributed to the Na^+^ extrusion in NaCl stressed roots (Sun et al., 2009a,b). H^+^-ATPase activity and the H^+^/ATP coupling ratio of PMs isolated from cucumber roots were enhanced by NaHS treatments (Janicka et al., 2017). Furthermore, NaHS (100 μM) stimulated protein abundance of PM H^+^-ATPase and the transcription of *HvHA1* and *HvSOS1* in barley roots (Chen et al., 2015). Therefore, the H_2_S-activated H^+^-pumps in the PM maintain electrochemical H^+^ gradients, which in turn promote secondary Na^+^/H^+^ antiport across root PMs for both poplar species (Blumwald et al., 2000; Zhu, 2003). Notably, H_2_S induced enhancement of Na^+^/H^+^ was more pronounced in the salt-sensitive poplar relative to the salt-resistant *P. euphratica* (**Figures 3**, **4**). The salt-resistant poplar exhibited typically a higher capacity for Na^+^/H^+^ exchange in the absence of exogenous NaHS (**Figures 3**, **4**). This indicates that its PM Na^+^/H^+^ antiport system extrudes Na^+^ more effectively than that of the salt-sensitive poplar (Sun et al., 2009a,b). We have shown that the salt signaling network of H_2_O_2_, cytosolic Ca^2+^, extracellular ATP (eATP), and NO help mediate Na^+^ extrusion in *P. euphratica* (Sun et al., 2012; Zhang et al., 2015).

In this study, an apparent H^+^ efflux which indicates activity of H^+^-ATPase was not recorded in the NaHS-treated roots (**Figure 4**). Instead, H_2_S enlarged the influx of H^+^ in *P. popularis* roots (**Figure 4**). The H_2_S-enhanced H^+^ influx corresponding to Na^+^ efflux resulted from the Na^+^/H^+^ antiport, which was promoted by PM H^+^-ATPase (**Figures 3**, **4**, **6**). However, the NMT data only show a net flux of H^+^ across the PM in root cells that undergoing Na^+^/H^+^ exchange, instead of the unidirectional flux (Sun et al., 2009b). Using NMT microelectrodes, a net H^+^ influx was usually recorded in roots of salt-resistant poplar and mangroves after ST (24 h) and LT salt stress (from 7 to 21 days, Sun et al., 2009a,b; Lu et al., 2013). This indicates that the PM H^+^-pumps had already established a steep H^+^ gradient upon NaCl exposure, thus promoting the Na^+^/H^+^ antiport across the PM in these salt-resistant species. In accordance, H^+^ efflux promoted by the PM H^+^-ATPase was observed in salt shock treatment (40 min) in *P. euphratica* roots (Sun et al., 2009b). Therefore, the H_2_S-stimulated Na^+^/H^+^ antiport was presumably due to the upward-regulated H^+^-ATPases. Moreover, the effect of NaHS on H^+^ influx is more pronounced in no-salt *P. popularis* roots than in NaCl-treated ones (**Figure 4**). This is presumably due to the salt inhibition of NaCl on Na^+^/H^+^ antiport system in this salt-sensitive poplar. We have previously shown that NaCl treatment had an inhibitory effect on the Na^+^/H^+^ antiport (Sun et al., 2009a,b) and H^+^-ATPase activity for *P. popularis* (Ma et al., 2010).

### K^+^ Homeostasis

K^+^ flux profiles showed that H_2_S contributed to K^+^ homeostasis under NaCl stress in poplars. Salt shock, as well as short- and long-term salt stress resulted in a significant net K^+^ loss in both the salt-resistant *P. euphratica* and the salt-sensitive *P. popularis* (**Figures 1**, **2**). The results are consistent with previous studies of these species (Sun et al., 2009b). Together, these show that salt-induced K^+^ efflux is mediated by KORCs and NSCCs in poplar (Sun et al., 2009a,b) and herbaceous species (Shabala et al., 2005, 2006; Chen et al., 2007; Shabala and Cuin, 2008). H_2_S significantly reduced K^+^ loss evoked by ST and LT salt stress applied to the two species (**Figure 2**). H_2_S can similarly enhance salt tolerance by preventing K^+^ loss in salt-treated *M. sativa* (Lai et al., 2014) and barley (Chen et al., 2015). A more pronounced reduction of K^+^ efflux induced by NaHS was observed in the LT salt stressed *P. popularis* roots compared to those of *P. euphratica* (**Figure 2**). The high flux rates of K^+^ in *P. euphratica* roots reveal the high concentration of K^+^ sources under LT salt stress (Sun et al., 2009b). We previously showed that relative to salt-sensitive species, *P. euphratica* had a higher capacity to maintain K^+^ uptake and transport under saline conditions (Chen et al., 2001, 2003). The NaCl-induced K^+^ efflux was inhibited by TEA in *P. popularis*, but significantly increased by sodium vanadate for both poplars studied under conditions of LT salt stress and NaHS treatment (**Figure 5**). This indicates that NaCl-induced K^+^ loss is through depolarization-activated K^+^ channels (Chen et al., 2007; Shabala and Cuin, 2008). We infer that the H_2_S-decreased K^+^ loss likely results from activated H^+^-pumping activity in the salinized roots. The activated H^+^-ATPase may inhibit the NaCl-depolarized membrane potential, thus lessening K^+^ flow through PM-localized DA-KORCs/NSCCs in *P. euphratica* and *P. popularis* roots (Sun et al., 2009b). Furthermore, the activated H^+^-ATPase might contribute to the H_2_S-induced K^+^ influx in non-salinized roots, in particular *P. euphratica* (**Figure 2**). NaHS activated H^+^-ATPase in the PM, which could produce a steep H^+^ gradient, thus promoting the entry of K^+^ via hyperpolarization-activated potassium channels (Ilan et al., 1996; Sun et al., 2009b).

### Species Difference in the Response to Salt and H_2_S

Our data showed that the response to salt and H_2_S varied between *P. euphratica* and *P. popularis*. NaCl caused a remarkable K^+^ loss but a less Na^+^ extrusion in the salt-sensitive poplar (**Figures 2**, **3**). Compared to *P. popularis*, the salt-resistant *P. euphratica* exhibited a pronounced effect to extrude Na^+^ and to retain K^+^ under salt stress (**Figures 2**, **3**). This is mainly due to its active PM Na^+^/H^+^ antiporters and H^+^ pumps in the PM (Sun et al., 2009a,b, 2010a,b). It is noting to find that H_2_S was more effective to assist *P. popularis* to retain K^+^/Na^+^ homeostasis under NaCl stress, as compared to the salt-resistant poplar. This is similar to our previous finding that Ca^2+^ application showed a more pronounced beneficial effect for salt-sensitive poplar to retain ionic homeostasis (Sun et al., 2009b). The difference in the response to salt between contrasting poplars is probably related to their differences in the production of salt signals. The salt-sensitive species, *P. popularis*, is insensitive to the salinity and unable to produce stress signals, such as H_2_O_2_ and NO, leading to uncontrolled conditions of ion toxicity and oxidative damage (Sun et al., 2010b). In contrast to *P. popularis*, NaCl salinity caused a rapid increase of H_2_O_2_ and NO in *P. euphratica* cells (Sun et al., 2010b). The salt-elicited H_2_O_2_ and NO enable *P. euphratica* cells to regulate ionic and ROS (reactive oxygen species) homeostasis under salinity stress in the longer term (Sun et al., 2010b). In this study, it is likely that the signal molecule H_2_S contribute to K^+^/Na^+^ homeostasis control in *P. popularis* through activations of both Na^+^/H^+^ antiporter and H^+^-ATPase in the PM. The beneficial effect of H_2_S was less pronounced in the salt-resistant poplar, due to the abundance of endogenous salt signals. The salt-elicited endogenous signals, such H_2_O_2_, cytosolic Ca^2+^, eATP, and NO, mediated ionic homeostasis in *P. euphratica* (Sun et al., 2010a,b, 2012; Zhang et al., 2015; Zhao et al., 2016), thus weakening the effect of exogenously applied H_2_S.

## Conclusion

NaHS treatments increased *P. euphratica* and *P. popularis* capacity to maintain root K^+^/Na^+^ homeostasis under saline conditions. NaHS application effectively limited the NaCl-induced K^+^ loss and simultaneously increased Na^+^ extrusion capacity. Beneficial effects of H_2_S are presumably due to the upward-regulated H^+^-ATPases, which (1) sustain the H^+^ gradient that drives the Na^+^/H^+^ antiport across the PM, and (2) preserve a less-depolarized membrane potential, which restricts K^+^ loss through depolarization-activated KORCs and NSCCs in the PM. As a result, K^+^/Na^+^ homeostasis in root cells is maintained in *P. euphratica* and *P. popularis* subjected to salinized conditions. The effect of H_2_S was more pronounced for roots of the salt-sensitive *P. popularis* subjected to higher NaCl conditions. This indicates that H_2_S has considerable potential for improving salt tolerance in salt-sensitive trees. The molecular mechanisms resulting in different signal transduction responses for both species require further investigation.

## Author Contributions

NZ, HpZ, and SC conceived of the original research project and selected methods. SC supervised the experiments. NZ, HpZ, HlZ, JS, CD, and YZ performed most of the experiments. JZ, RZ, XZ, and CL provided technical assistance to NZ, HpZ, HlZ, JS, CD, and YZ. NZ and HpZ designed the experiments and analyzed the data. NZ and HpZ refined the project and wrote the manuscript with contributions from all the authors. SL and SC revised the writing. All authors have read and approved the manuscript.

## Conflict of Interest Statement

The authors declare that the research was conducted in the absence of any commercial or financial relationships that could be construed as a potential conflict of interest.

## Footnotes

1 http://youngerusa.com

2 http://xbi.org
